# Outcome evaluation for the treatment of low flow venous and lymphatic malformations

**DOI:** 10.1186/s42155-024-00493-z

**Published:** 2024-11-29

**Authors:** R. M. Moussa, A. O. Oseni, S. Patel, L. Mailli, R. Morgan, L. A. Ratnam

**Affiliations:** 1 George’s Healthcare NHS Trust, St George’s University Hospitals NHS Foundation Trust, London, SW17 0QT UK; 2 School of Health & Medical Sciences, City St George’s, University of London, London, SW17 0RE UK

**Keywords:** Low flow venous and lymphatic malformations, Vascular malformation, Sclerotherapy

## Abstract

**Purpose:**

To propose a standardized method of subjectively and objectively evaluating outcomes of sclerotherapy in treating low flow vascular malformations.

**Materials and methods:**

Sixty-six patients with low flow vascular malformations (venous, lymphatic, or combined) were treated with percutaneous sclerotherapy using bleomycin, doxycycline, or sodium tetradecyl sulphate. Each lesion required between 2–5 sessions of sclerotherapy with 8-week intervals in between.

The success of sclerotherapy was evaluated subjectively and objectively. The subjective response was based on the degree of patient satisfaction, by recording improvement of their symptoms and quality of life. The objective response was based on the changes in lesion characteristics after treatment, by recording changes in size, sonographic features, number of cystic spaces, and development of phleboliths.

**Results:**

91% of our patients were satisfied with the treatment and reported improvement of symptoms and quality of life. Radiologically, 62% (41/66) of the patients had a reduction in lesion size, 77% (51/66) had a change in echogenicity, 84% (51/61) had a reduction in cystic spaces, and 68% (30/44) developed phleboliths. Of the patients reporting significant improvement, 94% displayed reduction in cystic spaces, 89% displayed change in the echogenicity and 71% showed changes in the size of the lesions, representing a linear correlation.

**Conclusion:**

Evaluating the outcomes of percutaneous sclerotherapy for treating vascular malformations is a recognized challenge. Creating a questionnaire with defined parameters to apply before and after treatment allows objective measurement of outcomes. This will enable improved treatment pathways and treatment choice for patients, informed consent, and enable outcome comparison with other centers.

**Supplementary Information:**

The online version contains supplementary material available at 10.1186/s42155-024-00493-z.

## Introduction

Vascular malformations are complex abnormalities causing significant morbidity and disability [1]. In 1996, the International Society for the Study of Vascular Anomalies (ISSVA) developed a classification system that has been significant in helping to clarify the distinctions between different vascular anomalies, leading to improved patient management (Table 1) [2, 3]. The most common vascular malformations are low flow vascular malformations (LFVM) which is the group of patients studied in this paper.

**Table 1:**
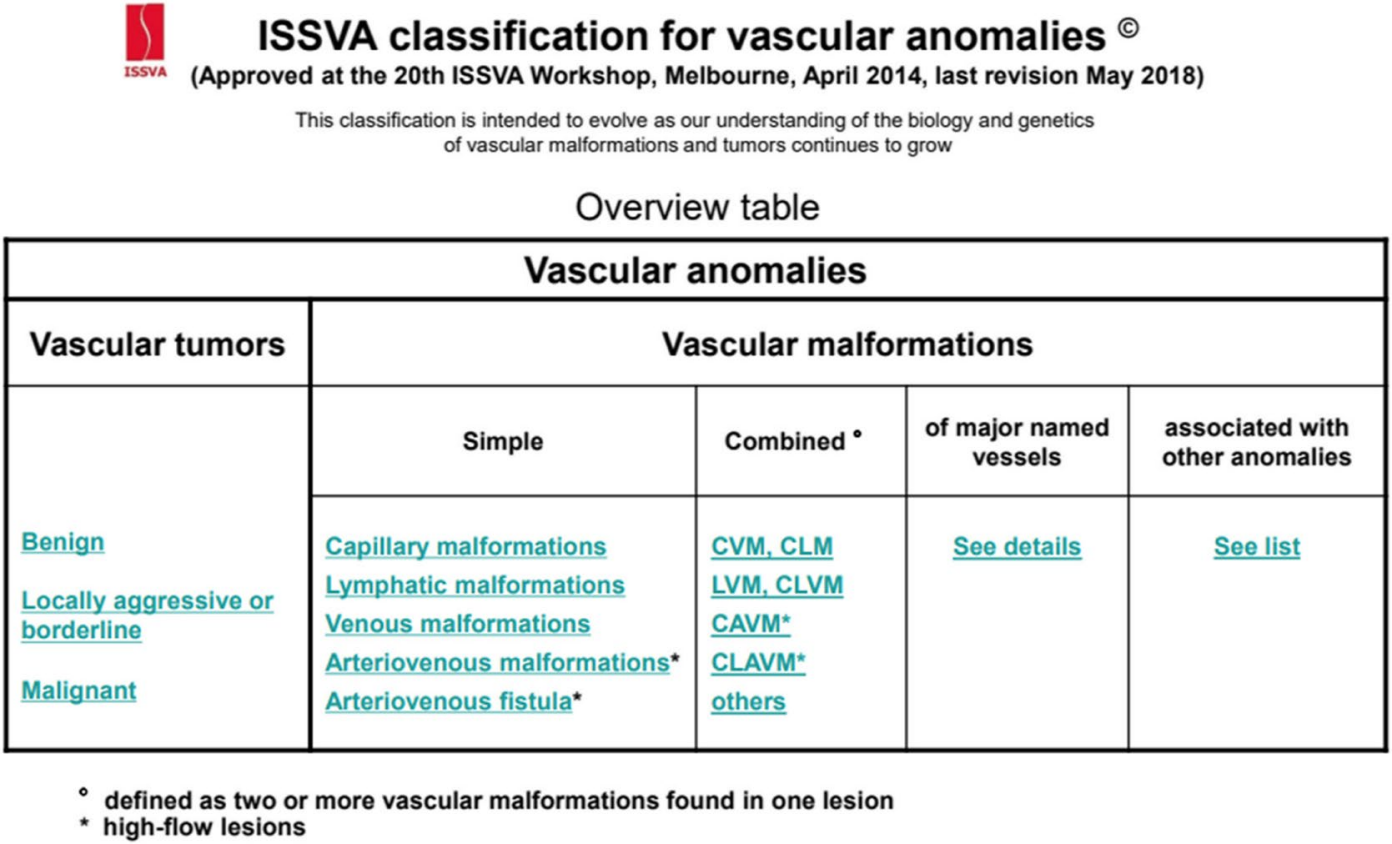
ISSVA Classification of vascular anomalies ©2018 International Society for the Study of Vascular Anomalies https://www.issva.org/classification [3]

LFVMs present with variable symptoms including swelling, pain, functional limitations, tissue ulceration and cosmetic issues [4]. Sclerotherapy is the treatment of choice for most symptomatic low flow venous and lymphatic malformations (VM and LM), usually requiring multiple treatment sessions to achieve a satisfactory response.

Publications have focused on the technical and short-term results of treatment, which have concluded that VMs are difficult to treat and that most patients require multiple therapeutic sessions for clinical effectiveness to be achieved [4–7]. Moreover, there is little consensus on the objective evaluation of treatment success of low-flow vascular malformations. Few research articles have described the improvement in symptoms and patient satisfaction as the key factor for assessment. However, there remains a need for more objective tools. A review of current literature confirms that there are still no definite criteria to evaluate sclerotherapy success [8].

The purpose of this study was to describe the outcome evaluation of the treatment of low flow malformations and to describe a standardized method that is both subjective and objective.

## Materials & methods

### Patients

**Fig. 1:**
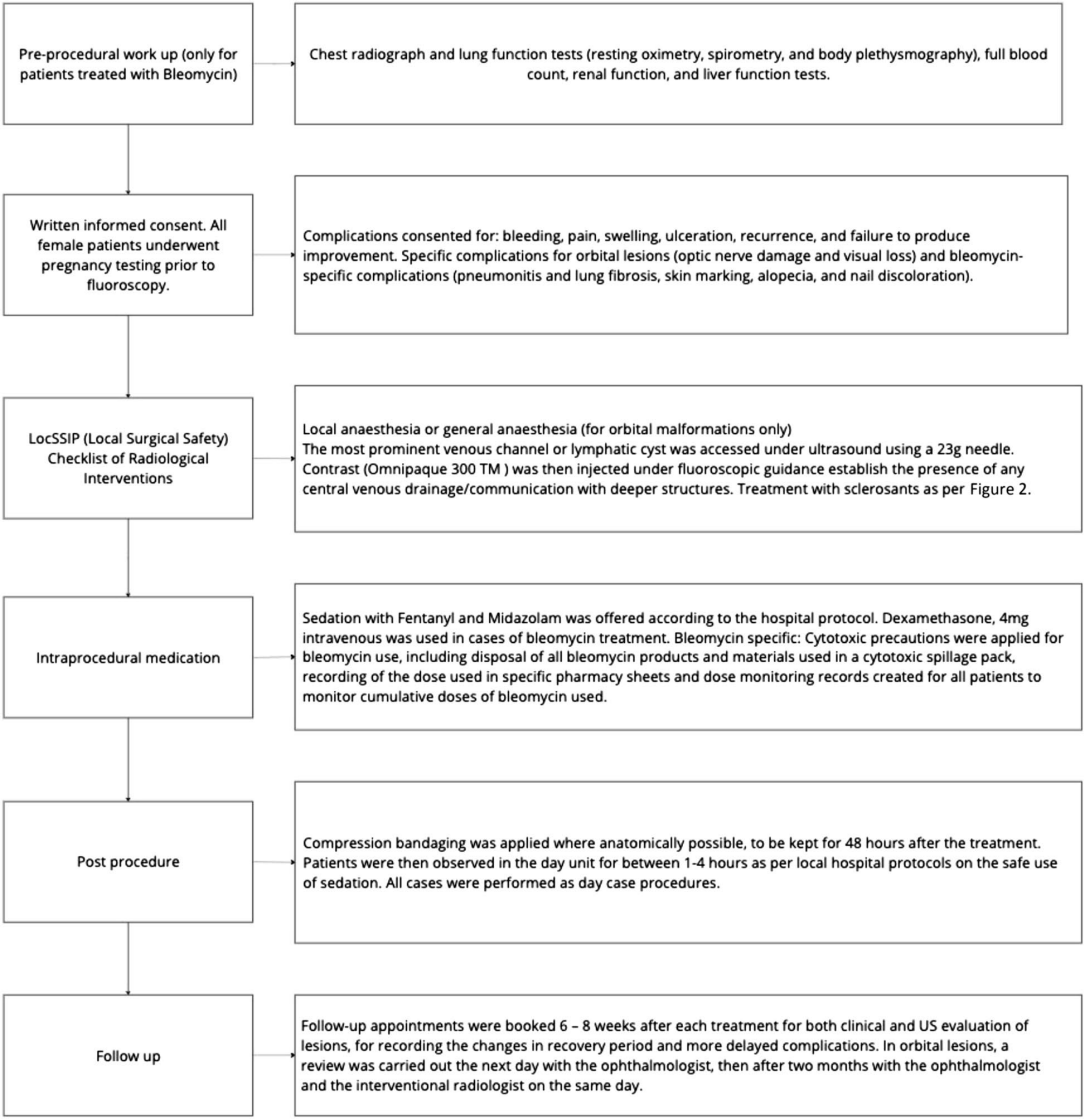
Treatment algorithm

**Fig. 2:**
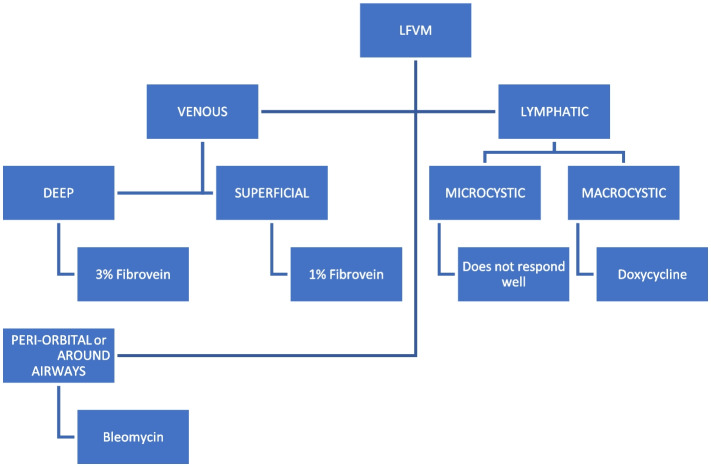
Choice of sclerosants. *LFVM – low flow vascular malformations

All patients were treated in our hospital over eight years (February 2012 to September 2020). Sixty-six patients with low flow venous, lymphatic, and mixed malformations treated from February 2012 to September 2020 were included. Data was collected by review of the electronic patient records, Radiology Information System and the Picture Archiving and Communication systems. The presence of high flow arterio-venous malformation was our only exclusion criterion. Clinical and radiological evaluation, treatment algorithm and choice of sclerosant have been summarised in Table 2, Figs. 1 and 2.

**Table 2:** Clinical and radiological evaluation

**Clinical symptoms**
Pain, swelling, cosmetic concerns, bruising, bleeding, change in appearance or size, itching, variation with weather, recurrence of symptoms after previous treatment, and any impairment of quality of life.
**Clinical signs**
Site, size, appearance, overlying skin discoloration or ulceration, and compressibility.
**Radiological assessment**
MRI sequences
T1WI, T2WI, fluid sensitive fat suppressed sequences in pre and post Gadolinium contrast injection. Accurate lesion extensions and measurements (in three dimensions), and characteristic signal intensity were recorded. Specific parameters: TR 425 and TE 9.912 for T1WI, TR 1800 and TE23.52 for PD Fat SAT, TR 5.544 and TE 1.692 for FGRE, TR 1800 and TE 43.968 for PD, TR 3000 and 101.587 for T2WI, and TR 640 and TE 22 for GR.
Ultrasound features
Compressibility, presence of phleboliths, number of cystic areas present and the size of the largest cyst or vascular channels.

### Response to treatment

The success of treatment was assessed subjectively and objectively. These data were recorded and compared before and after treatment, for each patient, separately (Appendix A-D).

For the subjective method, we asked all patients about their satisfaction with the treatment response, the improvement of their symptoms, and their quality of life. Questionnaires were completed by the patients before the procedure, for each treatment. The post-procedure questionnaires were completed after the procedures at the time of clinical review at 6–8 weeks post-treatment. These were recorded and classified into two categories: significant improvement of the symptoms, and mild or no improvement at all.

The objective assessment was done with ultrasound criteria documented post-treatment: change in the echogenicity, decrease in the number of cystic spaces, decrease in the size of the lesion (change by 5 mm- in at least one of the three diameters, was considered a true change in size rather than inter-operator variation in measurements), and development of thrombosis or phleboliths.

Further treatments were arranged if the patient was still experiencing symptoms and where there were remaining cystic areas or venous channels to be targeted in the next treatment. If symptoms had resolved patients were discharged and advised to seek re-referral if symptoms recurred in the future. Patients who were still not satisfied with the treatment response were discussed in the Lymphatic and Arteriovenous Anomaly (LAVA) multidisciplinary team (MDT) meeting to offer them surgical treatment options when applicable.

For statistical analysis, we used BM SPSS statistics (V. 27.0, IBM Corp., USA, 2020) for data analysis. Data were expressed as both numbers and percentages for categorized data.

The following tests were done:

1. Chi-square test to study the association between each 2 variables or comparison between 2 independent groups as regards the categorized data. The probability of error at 0.05 was considered sig., while at 0.01 and 0.001 are highly sig.
2. Diagnostic validity test: It includes % agreement and % disagreement.

## Results

This study included 66 patients; their ages ranged from one year to 75 years. Patients presenting symptoms included pain (52%), swelling (45%), cosmetic concerns (21%), impact on quality of life (19%), recurrence after previous treatment of any type (14%), bleeding (12%), bruising (4%), headaches (3%), blurred vision (3%) and pruritis (1%). The most common presenting symptom was pain while the least common was pruritis. Lesion location was classified into; lesions in the head and neck (44%), trunk (4%), lower limb (30%), upper limb (15%) and groin (7%). According to the lesion type, 57% of lesions were pure venous, 19% pure lymphatic, and 24% combined. These lesions were diagnosed mostly by US. Some lesions were diagnosed by MRI, in the first instance, which accounted for 24% of patients.

Most patients (75%) were reviewed and discussed in our LAVA MDT for optimal management planning, often including a combination of sclerotherapy and compression garments.

The sclerotherapy protocol as described (Fig. 1) was used in all patients. The number of sessions needed ranged from one session to a maximum of twelve sessions. However, most patients (95%) required 2–5 sessions, and approximately 4% required 6–8 sessions. The sclerosants used in treatment were Sodium tetradecyl sulphate (STS) in 71% of lesions, Bleomycin in 17%, and doxycycline in 16% of the lesions.

The standardized CIRSE classification system for complications was used to record our complications. We categorized complications into two groups: Group 1 no additional therapy or sequelae and Group 2 overnight admission but no additional therapy or sequelae [9]. In our study, we recorded complications in 45% of patients (30/66). All the complications were categorized as Group 1, apart from one complication (reaction to the sclerosant material) was considered Group 2. Our recorded minor complications (Group 1) included bruising, itching, swelling, pain, hyperpigmentation, small bleeding, ulceration, and numbness. Most of the complications were swelling and pain post-operatively, which we considered to be expected post-procedure response, and patients were educated about them during the consent before the procedure, and before discharge. Mucosal ulceration was documented in two patients, both were treated with STS sclerosant. This complication is also well documented in the literature and the probability of its occurrence was discussed as part of the informed consent process. All these reactions subsided within ten days without any further need for medical intervention or hospitalization (Group 1). The most common long-term complication was recurrence which is recognised as a feature of LFVMs.

One of our patients had an allergic reaction to the sclerosant, which presented immediately post-procedure with rigors and was treated immediately according to the hospital protocol. He was admitted for further observation overnight, with no additional treatment needed (Group 2).

Regarding sclerotherapy success, we measured the response based on subjective and objective parameters. Subjectively, 91% of our patients reported improvement in their symptoms in comparison to, 9% (6/66) who reported no improvement.

Objectively, we measured the response based on the changes in the size, echogenicity, and cystic spaces in the malformation after sclerotherapy. Regarding the change in size, 62% (41/66) of lesions showed a change in the size of lesions after sclerotherapy, while 38% (25/66) displayed no change. In terms of change in echogenicity, most of the lesions, about 77% (51/66), displayed a change in echogenicity, with most becoming more hyperechoic after treatment, while only 23% (15/66) showed no change. The number and size of cystic spaces were reduced after treatment in 84% (41/51) of the lesions. In venous-containing malformations, about 66% (30/44) of venous lesions developed phleboliths after sclerosant therapy.

**Fig. 3:**
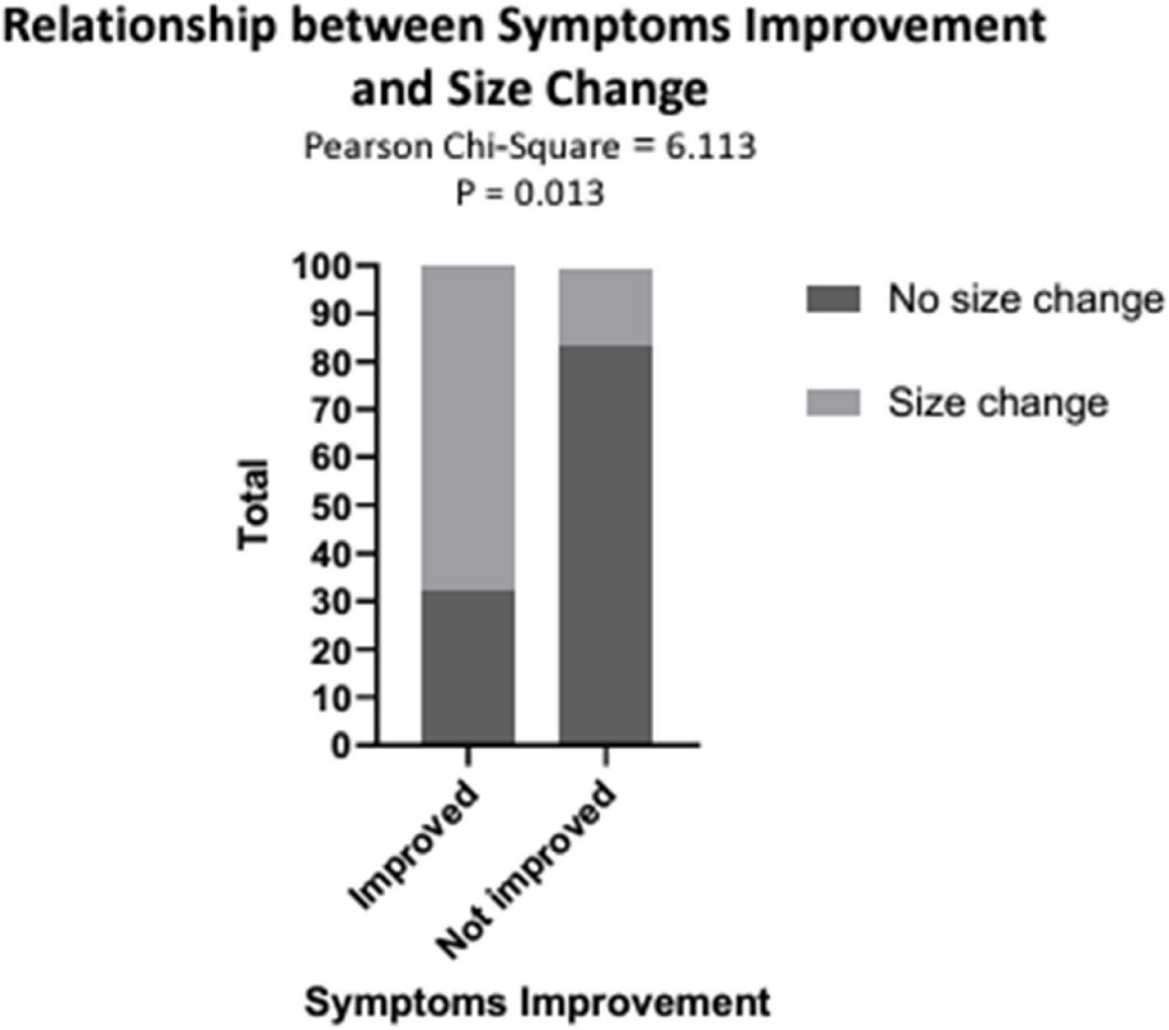
Shows the relationship between the improvement of patient symptoms and the change in size

Comparing the subjective and objective methods of evaluation; concerning the change in the size of the lesions, there is significant agreement between size and symptom improvement; among those improved (*n* = 59, 32.2% had no change in size, while 67.8% were associated with a change in size). On the other hand, of those who did not improve, the majority did not show any change in size (*n* = 6, 83.3% with no change in size while 16.7% had a change in size) (Table 3 and Fig. 3).

**Table 3:** Shows the relationship between the improvement of patient symptoms and the change in size

Change in the size after sclerotherapy					
			**Rating post-treatment**		**Total**
			**Significantly improved**	**Not improved**	
**Size change**	**no**	**Number of patients**	19	5	24
		**%**	32.2%	83.3%	36.9%
	**yes**	**Number of patients**	40	1	41
		**%**	67.8%	16.7%	63.1%
**Total**		**Number of patients**	59	6	65
		**%**	100.0%	100.0%	100.0%
		**Value**	***P***		
**Pearson Chi-Square**		6.113	.013		
**% Agreement**		= (40 + 5) / (65) = 69.2			
**% Disagreement**		= (19 + 1) / (65) = 30.8

**Fig. 4:**
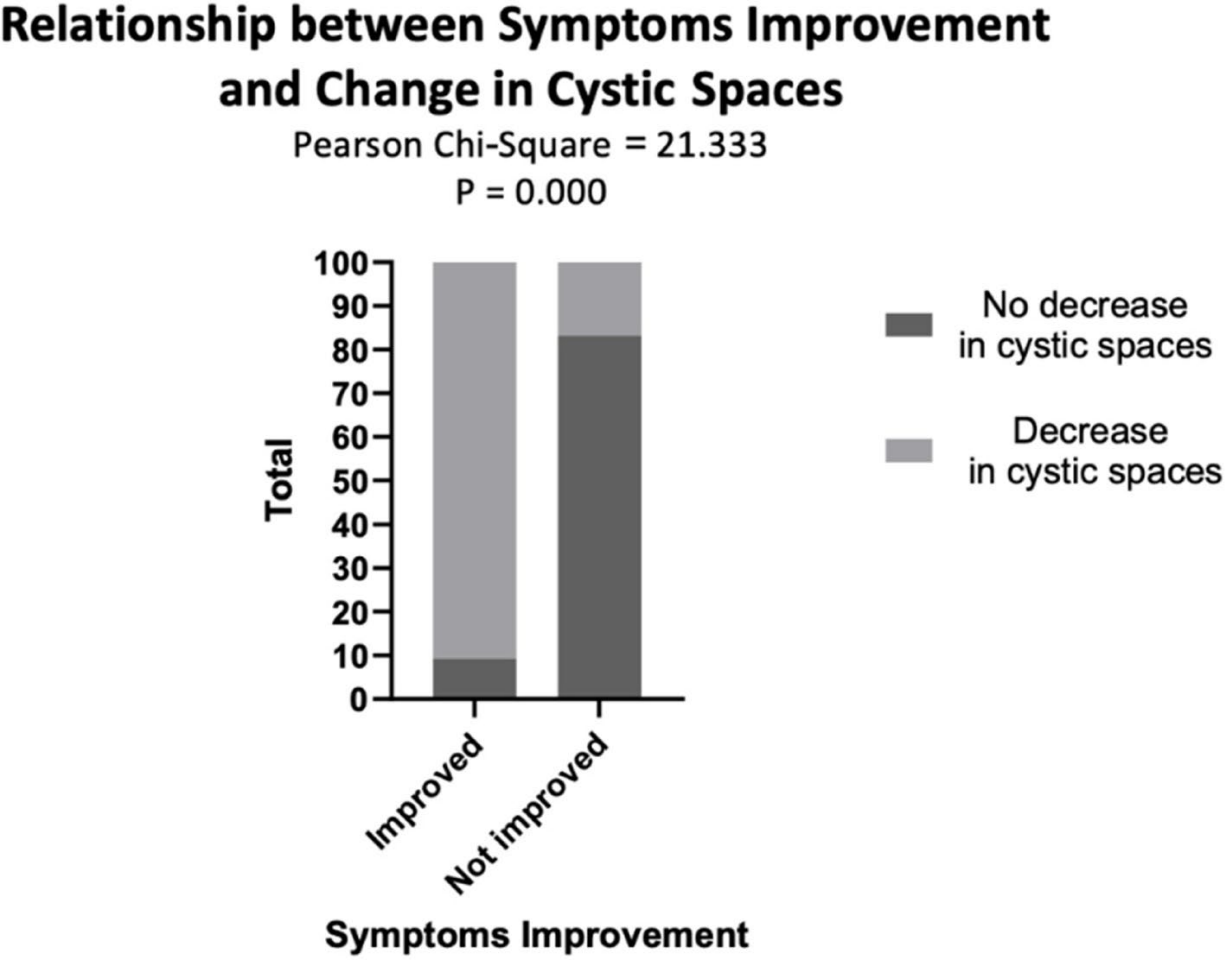
Shows the relationship between changes in cystic spaces and patient satisfaction

When comparing the decrease in cystic spaces and patient satisfaction, there is a highly significant agreement between the decrease in the cystic spaces in patients who reported significant improvement in their symptoms. Among those who reported significant improvement (*n* = 54), 9.3% had no decrease in the cystic spaces while 90.7% were associated with a decrease in the cystic spaces after sclerotherapy. On the other hand, for those who reported no improvement (*n* = 6), 83.3% had no decrease in the cystic spaces while 16.7% showed a decrease in cystic spaces (Table 4, Fig. 4).

**Table 4:** Shows the relationship between the change in cystic spaces and patient satisfaction

Change in cystic spaces after sclerotherapy					
			**Rating post-treatment**		**Total**
			**Improved**	**Not Improved**	
**Decrease in cystic spaces**	**no**	**Number of patients**	5	5	10
		**%**	9.3%	83.3%	16.7%
	**yes**	**Number of patients**	49	1	50
		**%**	90.7%	16.7%	83.3%
**Total**		**Number of patients**	54	6	60
		**%**	100.0%	100.0%	100.0%
	**Value**	***P***			
**Pearson Chi-Square**	21.333	.000			
**% Agreement**	= (49 + 5) / (65) = 83.1				
**% Disagreement**	= (5 + 1) / (65) = 9.2

**Fig. 5:**
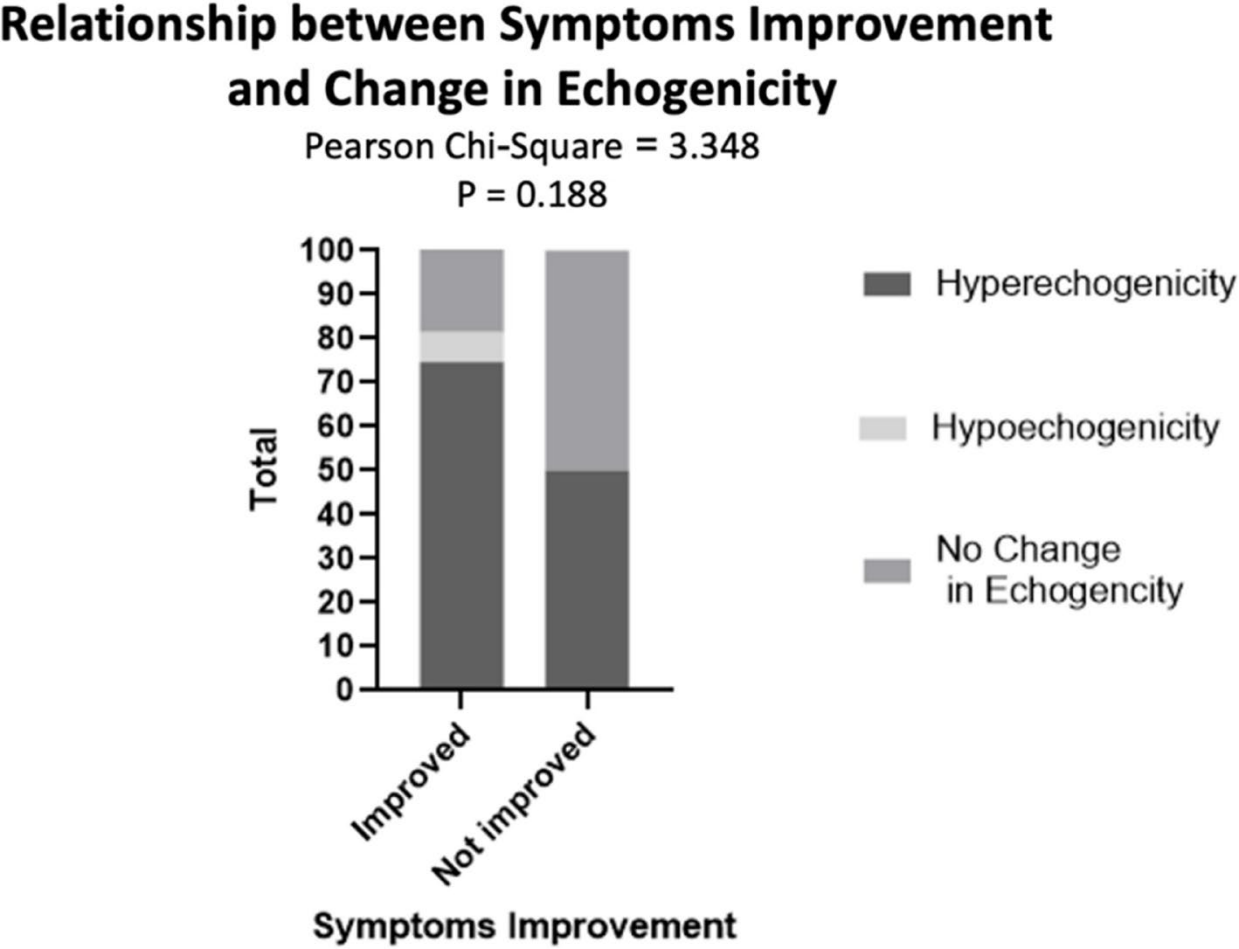
The relationship between the change in echogenicity and symptom improvement (non-significant)

Finally, with regards to the echogenicity of lesions on ultrasound, 74.6% of patients who reported improvement had changes in the lesion’s echogenicity after sclerotherapy, while 50% of the non-responding lesions had changes in the echogenicity. Thus, there is a non-significant agreement between change in echogenicity and symptom improvement (Table 5, Fig. 5).

**Table 5:** Displays the relationship between the change in echogenicity and symptom improvement (non-significant)

Change in echogenicity and symptom improvement					
			**Rating post-treatment**		**Total**
			**Improved**	**Not Improved**	
**Change in echogenicity**	**Hyper-echogenicity**	**Number of patients**	44	3	47
		**%**	74.6%	50.0%	72.3%
	**Hypo-echogenicity**	**Number of patients**	4	0	4
		**%**	6.8%	0.0%	6.2%
	**no**	**Number of patients**	11	3	14
		**%**	18.6%	50.0%	21.5%
**Total**		**Number of patients**	59	6	65
		**%**	100.0%	100.0%	100.0%
**Chi-Square Tests**					
	**Value**	***P***			
**Pearson Chi-Square**	3.348	.188

According to the sclerosant material used for sclerotherapy, the overall complete response following sclerotherapy with any agent was 90% (54/60). The success rate with STS was 95% (40/42), with Doxycycline was 71.4% (5/7), and with Bleomycin was 84.6% (9/11).

## Discussion

Sclerotherapy has increasingly become the first line of treatment for low-flow vascular malformations [10]. The lack of a standardized means of evaluating outcomes in this cohort of patients remains a challenge. We have set out a reproducible method of evaluation that combines objective and subjective means for assessing the outcomes of treatment based on our clinical experience and review of existing literature. This has resulted in questionnaires for patients and clinicians with fixed criteria to be utilized in describing and evaluating the treatment success for these lesions.

To identify the best subjective and objective criteria for evaluating the success of sclerotherapy, baseline assessment of patient records and imaging over three years, a literature review, and comparison to previous studies were carried out [11].

We used the patient satisfaction with the treatment response and the improvement in their symptoms and quality of life, as our subjective method of evaluation. The degree of improvement of the patient's symptoms and quality of life were evaluated using a graded scale for accurate comparison of the symptom intensity before and after sclerotherapy. The response was divided into three categories: significant, mild, and no improvement of symptoms. In our study, 91% of patients reported improvement in their symptoms.

**Fig. 6:**
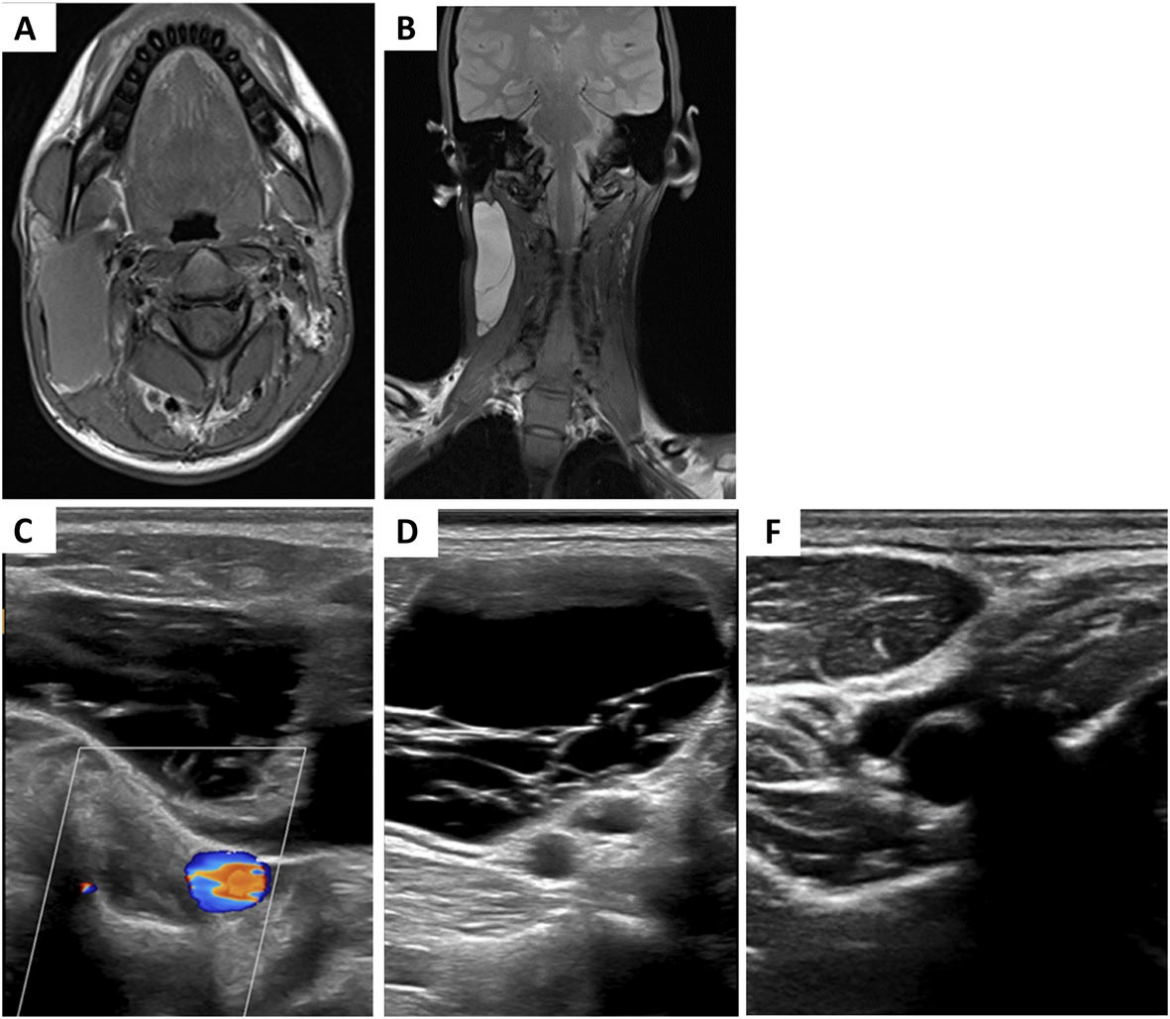
An 18-year-old female patient with a lymphatic malformation was treated with two sessions of sclerotherapy with doxycycline. The patient reported complete resolution of symptoms. **A** Axial T1WI image reveals well defined hypointense cystic lesion at right side of neck. **B** Coronal T2WI shows the lesion of high signal intensity. **C** Doppler US reveals no flow within the lesion in keeping with a low flow vascular malformation with normal flow within the right sided neck vessels. **D** US image displays multiple internal septations in the lesion. **E** Complete resolution of the lesion after two sessions of sclerotherapy

**Fig. 7:**
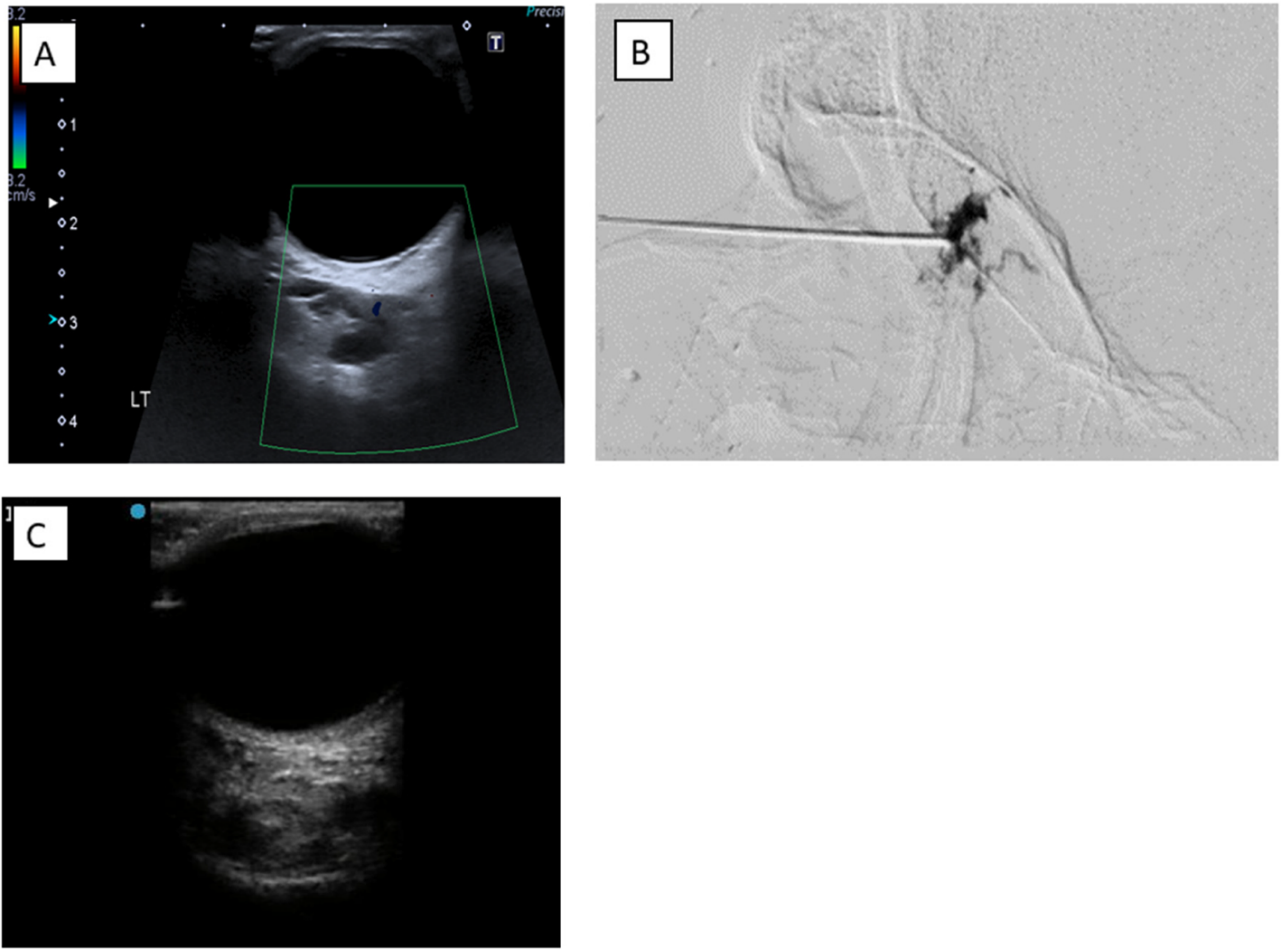
A 26-year-old female patient with a retroorbital low flow venous malformation in the deep aspect of the left orbit, treated with 2 sessions of Bleomycin sclerotherapy. The patient reported significant improvement in symptoms on her follow up after two months. **A** US image showing a lesion with multiple variable-size cysts and echogenic parenchyma. **B** Angiography images with contrast injected within the lesion prove that there is no communication with the cavernous sinus. **C** US image after sclerotherapy, revealing the complete disappearance of the cystic spaces

Objectively, we used the changes in lesion characteristics as indicators of sclerotherapy success. Each lesion was examined clinically and radiologically, described regarding its colour, covering skin, site, location, size, extent, echogenicity, type, flow by Doppler, presence of cystic spaces (with measurement of the largest cystic space, and follow-up of its size), and the presence of thrombosis or phleboliths. Most of the included lesions in the study displayed changes in the lesion characteristics after sclerotherapy (Figs. 6 and 7).

Our results compare favourably with other studies in the literature [4, 11–13]. For example, Van der Linden et al. [14] evaluated the results after percutaneous treatment of low-flow VMs and high-flow arterial VMs, with special interest in patient experience regarding clinical effect and overall patient satisfaction with the therapy, supposing that patient satisfaction is the most relevant outcome for this benign disorder. They found that clinical success, measured by partial or complete relief of symptoms, was reported by 38 (58%) of 66 patients with durable long-term results in most patients, and that clinical success was strongly related to patient satisfaction [4]. However, they recorded a rather large group of 28 (42%) patients, who had no clinical success 3 months after treatment. They noted that only patient satisfaction had been used to assess the outcome, while the effect of VM size, classification, or percutaneous treatment method chosen on clinical success was not assessed [4].

Another study published in the Canadian Association of Radiologists Journal in 2022 [13], correlated imaging findings post-sclerotherapy of low-flow vascular malformations with clinical outcome. They retrospectively correlated pre- and post-treatment sonographic volume with clinical outcomes in 29 patients who had sclerotherapy in their department over 14 years. Nineteen patients had both pre-treatment US and MRI, showing a correlation in volume between the 2 modalities (*P* < 0.001). Post-treatment change in volume correlated with clinical outcome for combined venous and LMs, with no correlation found when venous and lymphatic malformations were considered separately. The study concluded that ultrasound can potentially be used as an objective tool in evaluating sclerotherapy treatment response of low-flow vascular [13].

A further recent study published in 2023, compared the degree of patient satisfaction to the change in volumetric MRI size of lesions before and after treatment as an objective way to detect the effectiveness of sclerotherapy [12]. Outcome analysis of 45 patients treated with sclerotherapy showed good and excellent results after the last sclerotherapy was achieved in 36% and 29% of patients respectively, corresponding to a decrease of greater than 50% in 60% VM. Most patients did not require additional sclerotherapy due to near complete symptomatic relief even for patients who did not achieve a good response on MRI [12].

Our rate of complication was well within rates published in previous studies [9, 12, 15, 16]. For example, Van der Linden et al. [14], report a complication rate of 40% (27 of 66) of which the majority were minor [14]. Although they reported 12 (18%) of patients with major complications, these were all patients treated with ethanol or radiofrequency ablation, which is not used in our practice [4].

Finally, we compared our results with the systemic review and meta-analysis conducted by De Maria L, et al. which included 37 studies with a total of 2,067 patients with vascular malformations, all located in the head and/or neck region. The smallest study included 10 patients and the largest included 358 patients. The mean age was 24.9 years. The highest number of treated malformations per study was 358, while the lowest was 10. The mean number of treatment sessions per patient was 2.4. The overall efficacy rate of sclerotherapy was high with complete and partial cure rates of over 90%. Over 90% of patients reported satisfaction with the results of their sclerotherapy treatments and over 70% reported improvements in QoL [17].

Our study included 66 patients, with vascular malformations located in lesions in the head and neck (44%), trunk (4%), lower limb (30%), upper limb (15%), and groin (7%). The number of sessions needed ranged from one session to a maximum of twelve sessions. However, most patients (95%) required 2–5 sessions, and approximately 4% required 6–8 sessions. In our study, the overall success rate was 91% with 64% of patients reporting significant improvement of their symptoms (complete response), and 26% reporting mild improvement (partial response). On the other hand, 9% of patients reported no improvement in their symptoms [17].

Most included studies in the meta-analysis used a single sclerosing agent for each vascular malformation, while the remainder used a combination of them. They reported the lowest complete cure rate of 55.5% with STS and the highest cure rate of 82.9% with pingyangmycin. In our study, the success rate of STS was 95% (40/42), for Doxycycline was 71.4% (5/7), and for Bleomycin 84.6% (9/11). The lesions where more than one sclerosant was used were excluded. Direct comparisons of sclerosant efficacy are difficult due to multiple variables including lesion size, availability of different sclerosants, volume of sclerosant utilised and sclerotherapy technique which differ greatly from one centre to the next.

Our study is unique in its correlation between the subjective response to treatment and the objective response, in the form of clinical examination and multiple radiological parameters in the assessment of the treated lesions. In addition to the change in lesion size, the changes in echogenicity, number of cystic spaces, and thrombosis were also included (Appendix 1). On correlation, we found a highly significant association between the decrease in cystic spaces and the reported improvement in symptoms. There was also a significant association between the change in the size of the lesion after sclerotherapy and the reported improvement. However, there was no significant association between the change in echogenicity and the success of treatment.

Our study concluded that sclerotherapy using Doxycycline, Bleomycin, and STS, is a safe and highly effective treatment of low flow vascular malformation all over the body, including intra orbital lesions. The introduction of questionnaires to measure the subjective and objective responses to sclerotherapy has improved the evaluation of treatment success. The most reliable factors for treatment evaluation which correlate to symptom improvement are size reduction and reduction in cystic spaces in the treated lesion.

## Conclusion

Evaluating the outcomes of percutaneous sclerotherapy for the treatment of vascular malformations is a recognized problem within the field. Creating a questionnaire with defined parameters to be applied before and after treatment allowed us to obtain objective measurements of outcomes in our patients. Our study confirms sclerotherapy to be a safe and effective treatment for low flow vascular malformations. The ability to measure outcomes in a standardized manner will enable improved treatment pathways and treatment choices for patients, informing consent with local outcome figures as well as enabling comparison of outcomes and results across centres if utilized more widely.

## Supplementary Information

Supplementary Material 1: Appendix: A) First presentation questionnaire- Patient’ sheet.

Supplementary Material 2: Appendix: B) Follow up questionnaire- Patient’s sheet.

Supplementary Material 3: Appendix: C) First presentation questionnaire- Doctor’s sheet.

Supplementary Material 4: Appendix: D) Follow up questionnaire- Doctor’s sheet. 

## Data Availability

Not applicable.

## Abbreviations

ISSVA: International Society for the Study of Vascular Anomalies
LFVM: Low Flow Venous Malformation
LM: Lymphatic Malformation
LocSSIP: Local Safety Standards for Invasive Procedures
MRI: Magnetic Resonance Imaging
US: Ultrasound
STS: Sodium tetradecyl sulphate
VM: Venous Malformation
