# Supplementary material for: Outcome evaluation for the treatment of low flow venous and lymphatic malformations
Source: CVIR Endovasc. 2024 Nov 29;7:84. doi: 10.1186/s42155-024-00493-z (PMC11607242; doi:10.1186/s42155-024-00493-z)
Supplement: Supplementary file 1 — Supplementary Material 1: Appendix: A) First presentation questionnaire- Patient’ sheet. [file 42155_2024_493_MOESM1_ESM.pdf]

EVALUATION QUESTIONNAIRE FOR TREATMENT OF LOW FLOW VENOUS AND LYMPHATIC MALFORMATIONS WITH PERCUTANEOUS SCLEROTHERAPY

HOSPITAL NUMBER: ……………………….

PATIENT'S NAME: ………………………………

DATE OF REVIEW: …………………………

FIRST PRESENTION QUESTIONNAIRE PATIENT'S SHEET

(PRE-TREATMENT)

(1) Please select your gender

- ☐ Male
- ☐ Female
- ☐ Other

(2) Which area of your body is affected?

- ☐ Head and Neck
- ☐ Trunk
- ☐ Lower Limb
- ☐ Upper Limb
- ☐ Groin
- ☐ Other (specify):…………………………

(3) PRESENTING SYMPTOMS

When did you first start noticing this lesion?

……………………………………………………………

(4) Which of these are the reasons for your visit today? (Tick all that apply)

- ☐ Pain
- ☐ Swelling
- ☐ Concerns regarding appearance
- ☐ Change in lesion over time
- ☐ Bleeding
- ☐ Bruising
- ☐ Headaches
- ☐ Blurred vision
- ☐ Itchy skin
- ☐ Fatigue
- ☐ Increase in size or discomfort with hot weather
- ☐ Recurrence of lesion or symptoms after treatment,
  please specify when was the last treatment………………………
- ☐ Other ………………………………………………………

EVALUATION QUESTIONNAIRE FOR TREATMENT OF LOW FLOW VENOUS AND LYMPHATIC MALFORMATIONS WITH PERCUTANEOUS SCLEROTHERAPY

HOSPITAL NUMBER: ....

PATIENT'S NAME: ....

DATE OF REVIEW: ....

FIRST PRESENTION QUESTIONNAIRE PATIENT'S SHEET

(PRE-TREATMENT)

(5) <u>SEVERITY OF SYMPTOMS:</u>

For the reasons selected above, please rate how distressing these symptoms are on a scale of 1 to 5 where five is "most distressing" and 0 is "no concern at all''

	0	1	2	3	4	5
Pain	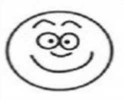 Does not hurt	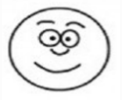 Hurts a Little	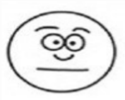 Hurts a little more	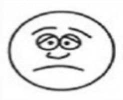 Hurts even more	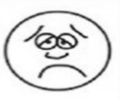 hurts a lot	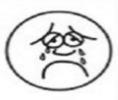 Hurts worse
Swelling						
Concerns regarding Appearance						
Change in Appearance over time						
Fatigue						
Bleeding						
Bruising						
Headaches						
Blurred Vision						
Itchy skin						

(OVAMA consensus, 2018)

(6) <u>OVERALL QUALITY OF LIFE:</u>

Please if this lesion is affecting the overall quality of your life, would you specify by choosing and rating the effect on a scale of 1 to 5 where five is "most distressing" and 0 is "no con cern at all".

	0	1	2	3	4	5
Mobility						
Activities of Daily living						
Work/Study						
Sports						
Leisure/Playing						
Confidence/self-esteem						
Emotional Wellbeing						

(OVAMA consensus, 2018)

EVALUATION QUESTIONNAIRE FOR TREATMENT OF LOW FLOW VENOUS AND LYMPHATIC MALFORMATIONS WITH PERCUTANEOUS SCLEROTHERAPY	HOSPITAL NUMBER: ....<br>PATIENT'S NAME: ....<br>DATE OF REVIEW: ....
FIRST PRESENTION QUESTIONNAIRE<br>PATIENT'S SHEET	(PRE-TREATMENT)

(7) **PATIENT EXPECTATIONS:**

Please describe your expected outcome following this treatment

……

……………………………………………………………………………………………..…………………

How many sessions do you think this treatment will include?

……..

Which of the following treatment outcomes would be your priority?

- ☐ Reduction of symptom severity
- ☐ Improvement of cosmetic appearance
- ☐ Improvement of the function (e.g. if it is affecting your mobility, functioning or work)

Do you expect recurrence of this lesion or your symptoms after treatment?

- ☐ Yes
- ☐ No
- ☐ Do not know
