# Supplementary material for: Outcome evaluation for the treatment of low flow venous and lymphatic malformations
Source: CVIR Endovasc. 2024 Nov 29;7:84. doi: 10.1186/s42155-024-00493-z (PMC11607242; doi:10.1186/s42155-024-00493-z)
Supplement: Supplementary file 2 — Supplementary Material 2: Appendix: B) Follow up questionnaire- Patient’s sheet. [file 42155_2024_493_MOESM2_ESM.pdf]

EVALUATION QUESTIONNAIRE FOR TREATMENT OF LOW FLOW VENOUS AND LYMPHATIC MALFORMATIONS WITH PERCUTANEOUS SCLEROTHERAPY

HOSPITAL NUMBER: ……………………..

PATIENT'S NAME: ……………………………..

DATE OF REVIEW: …………….……………

FOLLOW UP QUESTIONAIRE
PATIENT'S SHEET

POST-PROCEDURE
(SESSION………, TREATMENT………)

(1) RECOVERY PERIOD:

Please identify the length of your recovery period after the last procedure, and whether you had any of the following the responses. Please specify the duration of each:

- ☐ Length of recovery period, till return to full activity/ work (___ days)
- ☐ Pain (___ days)
- ☐ Swelling (___ days)
- ☐ Difficulty swallowing (___ days)
- ☐ Fever (___ days)
- ☐ Reduced mobility (___ days)
- ☐ Others (please specify): ………………………………………….

(2) COMPLICATIONS:

Please identify if you experienced any type of complication following the last treatment and its duration

- ☐ Bleeding (___ days)
- ☐ Ulceration (___ days)
- ☐ Infection/Abscess (___ days)
- ☐ Altered sensation (___ days)
- ☐ Other (please specify): ………………………………

Had you experienced any complication, please specify whether you underwent treatment for it

………

………

………

………

EVALUATION QUESTIONNAIRE FOR TREATMENT OF LOW FLOW VENOUS AND LYMPHATIC MALFORMATIONS WITH PERCUTANEOUS SCLEROTHERAPY

HOSPITAL NUMBER: ....

PATIENT'S NAME: ....

DATE OF REVIEW: ....

FOLLOW UP QUESTIONAIRE
PATIENT'S SHEET

POST-PROCEDURE
(SESSION.........., TREATMENT.........)

(3) EFFECTIVENESS OF TREATMENT:

Please describe the degree of improvement you feel in your symptoms since the treatment has been started

- ☐ Excellent Response (70%- 90% improvement)
- ☐ Moderate Response (50%- 70% improvement)
- ☐ Mild Response (20%- 50% improvement)
- ☐ No Response (0- 20% improvement)

(4) PATIENT'S SATISFACTION:

Could you identify to what extent are you satisfied with the treatment response? In other words, "What are the chances that you would take this treatment again?"

- ☐ Satisfied
- ☐ Dissatisfied

(5) PATIENT EXPECTATIONS:

Did the explanation and the leaflet you received following the first consultation gave you enough information about the treatment plan, outcomes and possibility of recurrence?

- ☐ Yes
- ☐ Somewhat
- ☐ No

Is there anything you wish you had been told that you weren't?

....

....

EVALUATION QUESTIONNAIRE FOR TREATMENT OF LOW FLOW VENOUS AND LYMPHATIC MALFORMATIONS WITH PERCUTANEOUS SCLEROTHERAPY

HOSPITAL NUMBER: ....

PATIENT'S NAME: ....

DATE OF REVIEW: ....

FOLLOW UP QUESTIONAIRE
PATIENT'S SHEET

POST-PROCEDURE
(SESSION.........., TREATMENT.........)

(6) SEVERITY OF SYMPTOMS:

Please rate how distressing your symptoms are, if you still have them, on a scale of 1 to 5 where five is "most distressing" and 0 is "no concern at all''

	0	1	2	3	4	5
Pain	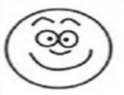 Does not hurt	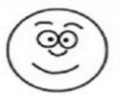 Hurts a little	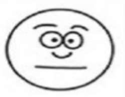 Hurts a little more	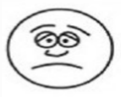 Hurts even more	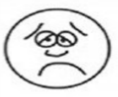 hurts a lot	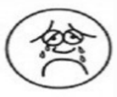 Hurts worse
Swelling						
Concerns regarding Appearance						
Change in Appearance over time						
Fatigue						
Bleeding						
Bruising						
Headaches						
Blurred Vision						
Itchy skin						

(OVAMA consensus, 2018)

(7) Overall Quality of Life improvement:

Please if this lesion is still affecting the overall quality of your life, would you specify by choosing and rating the effect on a scale of 1 to 5 where five is "most distressing" and 0 is "no con cern at all".

	0	1	2	3	4	5
Mobility						
Activities of Daily living						
Work/Study						
Sports						
Leisure/Playing						
Confidence/self-esteem						
Emotional Wellbeing						

(OVAMA consensus, 2018)
