# Supplementary material for: Outcome evaluation for the treatment of low flow venous and lymphatic malformations
Source: CVIR Endovasc. 2024 Nov 29;7:84. doi: 10.1186/s42155-024-00493-z (PMC11607242; doi:10.1186/s42155-024-00493-z)
Supplement: Supplementary file 3 — Supplementary Material 3: Appendix: C) First presentation questionnaire- Doctor’s sheet. [file 42155_2024_493_MOESM3_ESM.pdf]

EVALUATION QUESTIONNAIRE FOR TREATMENT OF LOW FLOW VENOUS AND LYMPHATIC MALFORMATIONS WITH PERCUTANEOUS SCLEROTHERAPY

HOSPITAL NUMBER: ………………………..

PATIENT'S NAME: ....

DATE OF REVIEW: …………….…………….

FIRST PRESENTATION QUESTIONNAIRE DOCTOR'S SHEET

(PRE-TREATMENT)

(1) Description of the lesion by clinical examination:

PARAMETER	LESION DESCRIPTION BEFORE TREATMENT
Site	
Number	
Size<br>(in two maximum dimensions)	
Color	
Consistency/ induration	
Compressibility	
Surface<br>(raised, ulcerated, surface bleeding or lymph leak)	

EVALUATION QUESTIONNAIRE FOR TREATMENT OF LOW FLOW VENOUS AND LYMPHATIC MALFORMATIONS WITH PERCUTANEOUS SCLEROTHERAPY

HOSPITAL NUMBER: ....

PATIENT'S NAME: ....

DATE OF REVIEW: ....

FIRST PRESENTATION QUESTIONNAIRE
DOCTOR'S SHEET

(PRE-TREATMENT)

(2) Description of the lesion by US:

PARAMETER	LESION DESCRIPTION BEFORE TREATMENT
Site and extension into deep tissue	
Number	
Size<br>(in three maximum dimensions)	
Main Echogenicity<br>(in comparison to related muscles)	☐ Hyper-echoic<br>☐ Iso-echoic<br>☐ Hypo-echoic
Consistency	☐ Solid<br>☐ Cystic<br>☐ Mixed solid-cystic
Sizes of the largest three cystic spaces in single maximum diameter (In cystic and mixed lesions)	
Presence of Thrombosis	☐ Yes ☐ No
Presence of Phleboliths	☐ Yes ☐ No
Compressibility	☐ Yes ☐ No
Flow by Color Doppler	☐ Yes ☐ No
Feeding artery or<br><br>Draining vein	☐ Yes ☐ No<br><br>☐ Yes ☐ No
Diagnosis of malformation	☐ Venous<br>☐ Lymphatic<br>☐ Veno-lymphatic
