# Supplementary material for: Outcome evaluation for the treatment of low flow venous and lymphatic malformations
Source: CVIR Endovasc. 2024 Nov 29;7:84. doi: 10.1186/s42155-024-00493-z (PMC11607242; doi:10.1186/s42155-024-00493-z)
Supplement: Supplementary file 4 — Supplementary Material 4: Appendix: D) Follow up questionnaire- Doctor’s sheet. [file 42155_2024_493_MOESM4_ESM.pdf]

EVALUATION QUESTIONNAIRE FOR TREATMENT OF LOW FLOW VENOUS AND LYMPHATIC MALFORMATIONS WITH PERCUTANEOUS SCLEROTHERAPY

HOSPITAL NUMBER: ………………………..

PATIENT'S NAME: ………………………………

DATE OF REVIEW: …………………………..

FOLLOW UP QUESTIONAIRE
DOCTOR'S SHEET

POST-PROCEDURE
(SESSION………., TREATMENT………)

(1) Changes of the lesion as observed clinically:

PARAMETER	LESION DESCRIPTION AFTER TREATMENT
Site	
Number	
Size<br>(in two maximum dimensions)	
Color	
Consistency/ induration	
Compressibility	
Surface<br>(raised, ulcerated, surface bleeding or lymph leak)	

EVALUATION QUESTIONNAIRE FOR TREATMENT OF LOW FLOW VENOUS AND LYMPHATIC MALFORMATIONS WITH PERCUTANEOUS SCLEROTHERAPY	HOSPITAL NUMBER: ....<br>PATIENT'S NAME: ....<br>DATE OF REVIEW: ....
FOLLOW UP QUESTIONAIRE<br>DOCTOR'S SHEET	POST-PROCEDURE<br>(SESSION.........., TREATMENT.........)

(2) Changes of the lesion as observed by Ultrasound:

PARAMETER	LESION DESCRIPTION AFTER TREATMENT	
Site and extension into deep tissue		
Number		
Size<br>(in three maximum dimensions)		
Main Echogenicity<br>(in comparison to related muscles)	☐ Hyper-echoic<br>☐ Iso-echoic<br>☐ Hypo-echoic	
Consistency	☐ Solid<br>☐ Cystic<br>☐ Mixed solid-cystic	
Sizes of the largest three cystic spaces in single maximum diameter (In cystic and mixed lesions)		
Presence of Thrombosis	…Yes	..No
Presence of Phleboliths	…Yes	..No
Compressibility	…Yes	..No
Flow by Color Doppler	…Yes	..No
Feeding artery or<br><br>Draining vein	…Yes<br><br>…Yes	..No<br><br>..No
Diagnosis of malformation	☐ Venous<br>☐ Lymphatic<br>☐ Veno-lymphatic	
Sclerosant material used	☐ STS<br>☐ Doxycycline<br>☐ Bleomycin	
